# Population norms of health-related quality of life in Moscow, Russia: the EQ-5D-5L-based survey

**DOI:** 10.1007/s11136-020-02705-0

**Published:** 2020-11-25

**Authors:** Malwina Hołownia-Voloskova, Aleksei Tarbastaev, Dominik Golicki

**Affiliations:** 1 Department of Experimental and Clinical Pharmacology, Medical University of Warsaw, Banacha 1b St, 02-097 Warsaw, Poland; 2 Health Technology Assessment Division, State Budgetary Institution “Research Institute for Healthcare Organization and Medical Management of Moscow Healthcare Department”, Moscow, Russia; 3 Department of Educational Programs Development, Center for Health and Social Development, Yury Luzhkov Moscow Metropolitan Governance University, 28 Sretenka St, Moscow, Russian Federation 107045

**Keywords:** Health-related quality of life, Patient-reported outcomes, Normative values, Reference values, EQ-5D, Russia

## Abstract

**Purpose:**

To develop population norms for the EQ-5D-5L questionnaire based on a representative sample of Moscow citizens.

**Methods:**

We used quota sampling accounting for sex, age group and administrative district of residence. Respondents in randomly selected outdoor and indoor locations were surveyed with the official Russian paper-and-pencil version of the EQ-5D-5L questionnaire and a set of socio-demographic questions. We estimated four types of EQ-5D results: the distribution of limitations according to EQ-5D-5L dimensions, the perception of the health-related quality of life (HRQoL) with a visual analogue scale (EQ VAS), the unweighted score for a respondent’s health state (Level Sum Score, LSS) and the Russian health preferences-based weighted score (EQ index). In order to estimate the EQ-5D-5L index, we used a newly developed Russian EQ-5D-3L value set, together with EuroQol Group cross-over methodology.

**Results:**

A total of 1020 respondents (18–93 years old) from the general Moscow adult population completed the EQ-5D-5L questionnaire. HRQoL domains with the largest number of identified health limitations were pain/discomfort (48.6%) and anxiety/depression (44.1%). Two hundred seventy-nine respondents (27.0%) did not report any health restrictions. The mean EQ VAS and EQ-5D-5L index were 74.1 (SD 17.3) and 0.907 (0.106) respectively. Multivariate analysis showed that female sex, advanced age and lack of access to the Internet had a negative influence on HRQoL, whereas residence in certain districts had a positive impact.

**Conclusions:**

The study provides population norms of health-related quality of life in Moscow, measured according to the EQ-5D-5L questionnaire. These reference values can be used to optimise the effectiveness of resource allocation in healthcare.

**Electronic supplementary material:**

The online version of this article (10.1007/s11136-020-02705-0) contains supplementary material, which is available to authorised users.

## Introduction

Over the past few years, health technology assessment (HTA) has been implemented in most Central and Eastern European (CEE) countries—based on international standards or through developments to the original HTA system, as in post-Soviet countries [1]. Russia, being the largest country in the world and the world’s ninth most populous country, implemented HTA into federal law in 2014 [2]. In spite of this, local healthcare decision-makers do not commonly use health-related quality of life (HRQoL) in outcome measurement and resource allocation.

When measuring HRQoL, one can choose between generic and disease-specific instruments [3]. Generic instruments focus on general health status, including physical, functional, and emotional domains. They apply to every health state and may be useful for comparisons between unrelated medical technologies. In some specific diseases, their use may be limited by a lack of sensitivity. One of them, the EQ-5D, is by far the most commonly used instrument for calculating utility scores, both in the CEE region [1] and all over the world [4]. The main advantages of EQ-5D are its widespread use, brevity and simplicity of administration [5]. Official versions are available for over 200 languages, so the data collected with EQ-5D can be successfully utilised for international comparisons. A five-level version of the EQ-5D (EQ-5D-5L) was developed, to improve the sensitivity of the original, three-level version (EQ-5D-3L), by adding two intermediate answer options to all of the dimensions [6, 7].

Population reference data for a specific country or region (sometimes called population norm data or simply population norms), can be used to compare profiles for patients with particular conditions with data for the average person in the general population from a similar age and gender group. Additionally, population norms enable comparison of the burden of the disease with the general population’s health, as well as the measurement of health inequalities [8].

Moscow has one of the largest municipal economies in Europe, which also accounts for more than one-fifth of the country’s gross domestic product. In Russia, Muscovites are in second place in terms of life expectancy, second only to inhabitants of the Republic of Ingushetia [9]. Although there is a long tradition of health surveys in Moscow, none of them has involved instruments from the EQ-5D family.

Our study aimed to develop population norms for HRQoL in Moscow, based on the EQ-5D-5L questionnaire. Bearing in mind the size of Russia, and knowing that most of the pilot projects are carried out firstly in Moscow before encompassing other regions, we have decided to start from the capital.

## Methods

### Sample

To make our study representative, we used quota sampling. Quotas accounted for sex, age group and the district of residence (*okrug*). The official division of Moscow in place until 2012, with ten administrative areas, was used. Seven age strata were distinguished (18–24, 25–34, 35–44, 45–54, 55–65, 64–75 and 75+ years). Quotas were built based on the Russian Statistics Office (ROSSTAT) data from 2014 [10].

### Survey

The official Russian paper-and-pencil version of the EQ-5D-5L was used. The EQ-5D-5L consists of two parts: a descriptive system (a report of symptoms and abilities) and a visual analogue scale (EQ VAS). The descriptive system includes five dimensions: mobility (MO), self-care (SC), usual activities (UA), pain/discomfort (PD), and anxiety/depression (AD). EQ-5D-5L dimensions comprise five levels of severity: no problems, slight problems, moderate problems, severe problems, and extreme problems [4]. Responses for all the five dimensions can be combined to form a five-digit number describing the respondent’s health state (from “11111”, meaning “no problems at all”, to “55555”, meaning “extreme problems” in all five dimensions). A total of 3125 possible health states are defined in this way. The EQ-5D health states may be converted into a single summary index by applying a formula that attaches values to each of the levels in each dimension (EQ Index) [11]. To estimate the EQ-5D-5L index, we used a newly developed Russian EQ-5D-3L value set [12], together with EuroQol Group cross-over methodology [13]. We also calculated unweighted Level Sum Scores (LSS) of the health states, a crude measure of severity of EQ-5D profiles, which may be a useful tool for performing comparisons among different populations.

The additional socio-demographic questionnaire included questions about: respondents’ age, level of education, monthly income, smoker/non-smoker and Internet access and usage. The question about the education provided nine possible answers. For data analysis, the responses were aggregated into three levels: primary (up to 9 years of education), secondary (up to 11 years of teaching, secondary vocational, incomplete higher) or higher education (bachelor’s degree, master’s degree or higher).

### Data collection

Two researchers (MH, AT) approached randomly selected respondents in randomly chosen locations, including parks, bus stops, transport hubs, car washes, malls, markets, restaurants, cafes and universities. The respondents were asked to fill in the EQ-5D-5L on their own but were guided by the interviewer if necessary. Answers to questions concerning demographic characteristics were collected by the interviewers. Items were read by the interviewer a maximum three times, and no additional comments were provided. A total of 10% of the interviews were subjected to data input quality control (the data of every tenth questionnaire in the paper form were verified with the data entered in the electronic database). Interviewers’ cross-validation was performed (MH carried out control of the data from the questionnaires entered by AT and vice versa).

### Analysis

We estimated four types of EQ-5D results: (1) the distribution of limitations according to EQ-5D-5L dimensions, (2) the perception of the health-related quality of life with EQ VAS, (3) the unweighted score for the respondent’s health state—the Level Sum Score (LSS), and (4) the weighted score for the health state—the EQ-5D-5L index. For continuous variables, we calculated the following descriptive statistics: mean, standard deviation and 95% confidence interval. Estimations were presented for the whole sample, as well as for the seven predefined age groups, in the EuroQol Group’s standardised format, to facilitate comparative research. We used multiple linear regression to examine the associations of socio-demographic characteristics with the EQ-5D-5L index and EQ VAS scores. All variables, including age, were entered into the models as categorical variables. Regression coefficients were presented together with information about the level of statistical significance. The analysis was carried out using Microsoft Excel and StatsDirect software, version 2.8.0 (StatsDirect Ltd, England).

## Results

From February to October 2017, a total of 1020 respondents from Moscow completed the EQ-5D-5L questionnaire. The sample approximated to the general adult Moscow population in terms of age, gender and district of residence (see Table 1 for details). The respondents were aged 18–93 years (mean 44.8, SD 19.0). The sample was characterised by a relatively high level of education (nearly 60% holding a university degree; only 0.5% with just primary level education). A substantial percentage of respondents (21.6%) refused to disclose their income.

**Table 1:** Study sample characteristics and comparison with Moscow general adult population (*n* = 11,815,393)

	Study sample *n* (%)	General adult population* %
*N*	1020 (100.0)	
Sex		
Female	555 (54.4)	54.0
Male	465 (45.6)	46.0
Age groups, years		
18–24	184 (18.0)	18.3
25–34	190 (18.6)	17.6
35–44	170 (16.7)	16.4
45–54	150 (14.7)	15.0
55–64	126 (12.4)	12.8
65–74	106 (10.4)	10.4
≥ 75	94 (9.2)	9.5
Moscow District		
Central	69 (6.8)	6.4
East	126 (12.4)	12.6
North	98 (9.6)	9.7
North-East	118 (11.6)	11.8
North-West	88 (8.6)	8.2
South	148 (14.5)	14.9
South-East	116 (11.4)	11.4
South-West	121 (11.9)	11.9
West	116 (11.4)	11.1
Zelenograd	20 (2.0)	1.9
Education		
Primary	6 (0.5)	6.4
Secondary	411 (40.5)	51.3
Higher	599 (59.0)	42.1
No education	0 (0)	0.2
Income, RUB		
≤ 15,000	102 (10.0)	[≤ 14,000 RUB]: 5.3
15,001–30,000	246 (24.1)	[14,000–27,000]: 17.1
30,001–50,000	174 (17.1)	[27,000–45,000]: 23.7
50,001–80,000	147 (14.4)	[45,000–60,000]: 14.7
> 80,000	131 (12.8)	[> 60,000 RUB]: 39.2
Refused	220 (21.6)	–
Smoking status (current)		
No	736 (72.2)	72.5
Yes	276 (27.1)	27.0
Refused	8 (0.8)	0.5
Internet usage		
Everyday	785 (77.9)	89.2
Minimum once a week	81 (8.0)	
No	142 (14.1)	10.8

*https://rosstat.gov.ru

Table 2 presents the results of the EQ-5D-5L descriptive system according to gender and age group. The health-related quality of life domains with the most significant number of identified health restrictions were pain/discomfort (48.6%) and anxiety/depression (44.1%), with the lowest being self-care (11.5%). The most significant number of severe and extreme health limitations was identified in the mobility dimension (3.7%), followed by anxiety/depression (3.2%).

**Table 2:** Prevalence of EQ-5D-5L responses by age group and gender (%)

Level	Mobility			Self-care			Usual activities			Pain/discomfort			Anxiety/depression		
	Total	Male	Female	Total	Male	Female	Total	Male	Female	Total	Male	Female	Total	Male	Female
All	*n* = 1020	*n* = 465	*n* = 555												
1	64.3	68.2	61.1	88.5	89.5	87.7	68.0	73.3	63.6	51.4	58.5	45.4	55.9	61.3	51.4
2	23.1	22.4	23.8	9.0	9.0	9.0	23.9	19.1	27.9	36.8	31.8	40.9	30.6	27.1	33.5
3	8.8	8.0	9.5	1.5	1.1	1.8	5.8	5.6	5.9	9.4	8.6	10.1	10.4	9.7	11.0
4	3.6	1.5	5.4	1.0	0.4	1.4	1.9	1.5	2.2	2.1	1.1	2.9	2.4	1.9	2.7
5	0.1	0.0	0.2	0.0	0.0	0.0	0.4	0.4	0.4	0.4	0.0	0.7	0.8	0.0	1.4
Any problems	35.7	31.8	38.9	11.5	10.5	12.3	32.0	26.7	36.4	48.6	41.5	54.6	44.1	38.7	48.6
18–24 years	*n* = 184	*n* = 85	*n* = 99												
1	87.0	88.2	85.9	95.7	98.8	92.9	76.6	82.4	71.7	62.0	80.0	46.5	54.3	71.8	39.4
2	12.0	9.4	14.1	4.3	1.2	7.1	19.6	14.1	24.2	33.7	17.6	47.5	34.2	23.5	43.4
3	1.1	2.4	0.0	0.0	0.0	0.0	1.6	1.2	2.0	2.2	2.4	2.0	9.2	3.5	14.1
4	0.0	0.0	0.0	0.0	0.0	0.0	1.1	1.2	1.0	1.6	0.0	3.0	1.6	1.2	2.0
5	0.0	0.0	0.0	0.0	0.0	0.0	1.1	1.2	1.0	0.5	0.0	1.0	0.5	0.0	1.0
Any problems	13.0	11.8	14.1	4.3	1.2	7.1	23.4	17.6	28.3	38.0	20.0	53.5	45.7	28.2	60.6
25–34 years	*n* = 190	*n* = 83	*n* = 107												
1	85.3	88.0	83.2	96.3	96.4	96.3	74.7	72.3	76.6	62.6	62.7	62.6	58.9	62.7	56.1
2	12.1	8.4	15.0	3.2	3.6	2.8	17.4	16.9	17.8	29.5	26.5	31.8	29.5	27.7	30.8
3	2.6	3.6	1.9	0.5	0.0	0.9	5.8	7.2	4.7	7.9	10.8	5.6	8.9	8.4	9.3
4	0.0	0.0	0.0	0.0	0.0	0.0	1.6	2.4	0.9	0.0	0.0	0.0	1.6	1.2	1.9
5	0.0	0.0	0.0	0.0	0.0	0.0	0.5	1.2	0.0	0.0	0.0	0.0	1.1	0.0	1.9
Any problems	14.7	12.0	16.8	3.7	3.6	3.7	25.3	27.7	23.4	37.4	37.3	37.4	41.1	37.3	43.9
35–44 years	*n* = 170	*n* = 76	*n* = 94												
1	74.7	80.3	70.2	95.9	98.7	93.6	81.2	88.2	75.5	60.0	68.4	53.2	54.7	60.5	50.0
2	18.8	15.8	21.3	4.1	1.3	6.4	15.9	6.6	23.4	36.5	28.9	42.6	35.9	28.9	41.5
3	6.5	3.9	8.5	0.0	0.0	0.0	2.9	5.3	1.1	2.9	1.3	4.3	8.8	10.5	7.4
4	0.0	0.0	0.0	0.0	0.0	0.0	0.0	0.0	0.0	0.6	1.3	0.0	0.0	0.0	0.0
5	0.0	0.0	0.0	0.0	0.0	0.0	0.0	0.0	0.0	0.0	0.0	0.0	0.6	0.0	1.1
Any problems	25.3	19.7	29.8	4.1	1.3	6.4	18.8	11.8	24.5	40.0	31.6	46.8	45.3	39.5	50.0
45–54 years	*n* = 150	*n* = 69	*n* = 81												
1	66.0	75.4	58.0	93.3	97.1	90.1	68.7	81.2	58.0	48.0	55.1	42.0	61.3	62.3	60.5
2	24.0	17.4	29.6	5.3	2.9	7.4	28.0	17.4	37.0	44.0	39.1	48.1	24.7	23.2	25.9
3	6.7	5.8	7.4	1.3	0.0	2.5	3.3	1.4	4.9	6.7	4.3	8.6	10.7	10.1	11.1
4	3.3	1.4	4.9	0.0	0.0	0.0	0.0	0.0	0.0	1.3	1.4	1.2	3.3	4.3	2.5
5	0.0	0.0	0.0	0.0	0.0	0.0	0.0	0.0	0.0	0.0	0.0	0.0	0.0	0.0	0.0
Any problems	34.0	24.6	42.0	6.7	2.9	9.9	31.3	18.8	42.0	52.0	44.9	58.0	38.7	37.7	39.5
55–64 years	*n* = 126	*n* = 59	*n* = 67												
1	53.2	64.4	43.3	89.7	91.5	88.1	65.9	71.2	61.2	45.2	55.9	35.8	60.3	66.1	55.2
2	32.5	27.1	37.3	8.7	6.8	10.4	27.8	23.7	31.3	43.7	37.3	49.3	29.4	27.1	31.3
3	11.9	6.8	16.4	0.8	1.7	0.0	5.6	5.1	6.0	9.5	6.8	11.9	7.1	6.8	7.5
4	2.4	1.7	3.0	0.8	0.0	1.5	0.8	0.0	1.5	1.6	0.0	3.0	3.2	0.0	6.0
5	0.0	0.0	0.0	0.0	0.0	0.0	0.0	0.0	0.0	0.0	0.0	0.0	0.0	0.0	0.0
Any problems	46.8	35.6	56.7	10.3	8.5	11.9	34.1	28.8	38.8	54.8	44.1	64.2	39.7	33.9	44.8
65–74 years	*n* = 106	*n* = 50	*n* = 56												
1	27.4	28.0	26.8	76.4	72.0	80.4	57.5	64.0	51.8	37.7	42.0	33.9	49.1	52.0	46.4
2	43.4	54.0	33.9	18.9	24.0	14.3	32.1	30.0	33.9	39.6	44.0	35.7	30.2	30.0	30.4
3	17.0	14.0	19.6	1.9	2.0	1.8	6.6	4.0	8.9	16.0	12.0	19.6	15.1	16.0	14.3
4	12.3	4.0	19.6	2.8	2.0	3.6	3.8	2.0	5.4	5.7	2.0	8.9	3.8	2.0	5.4
5	0.0	0.0	0.0	0.0	0.0	0.0	0.0	0.0	0.0	0.9	0.0	1.8	1.9	0.0	3.6
Any problems	72.7	72.0	73.2	23.6	28.0	19.6	42.5	36.0	48.2	62.3	58.0	66.1	50.9	48.0	53.6
75+ years	*n* = 94	*n* = 43	*n* = 51												
1	12.8	9.3	15.7	50.0	46.5	52.9	27.7	32.6	23.5	21.3	18.6	23.5	47.9	41.9	52.9
2	38.3	51.2	27.5	34.0	44.2	25.5	39.4	39.5	39.2	34.0	41.9	27.5	27.7	32.6	23.5
3	30.9	32.6	29.4	6.4	7.0	11.8	22.3	20.9	23.5	35.1	34.9	35.3	17.0	18.6	15.7
4	17.0	7.0	25.5	9.6	2.3	9.8	9.6	7.0	11.8	7.4	4.7	9.8	5.3	7.0	3.9
5	1.1	0.0	2.0	0.0	0.0	0.0	1.1	0.0	2.0	2.1	0.0	3.9	2.1	0.0	3.9
Any problems	87.2	90.7	84.3	50.0	53.5	47.1	72.3	67.4	76.5	78.7	81.4	76.5	52.1	58.1	47.1

**Fig. 1:**
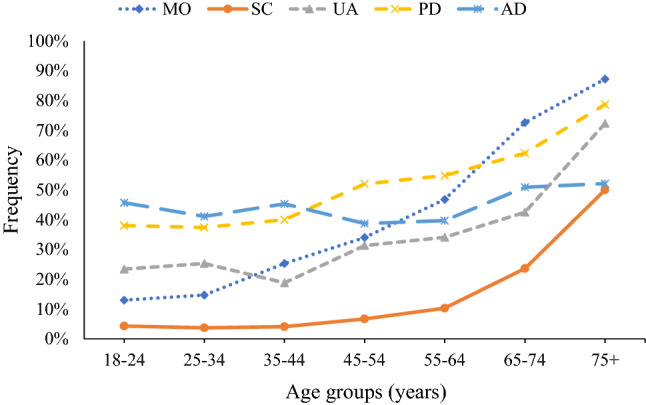
Prevalence of health limitations (any level of severity) for EQ-5D-5L dimensions according to age group

Health restrictions grew with age group (Fig. 1). This was particularly evident in terms of the mobility domain. The only dimension that followed a different pattern was anxiety/depression, where the level of limitations was generally high (> 40%) and relatively stable across age groups.

Analysing five EQ-5D-5L dimensions within seven age groups, we can state that, in general, females are characterised by expressing a lower health-related quality of life (in 29 out of 35 comparisons). The most striking difference concerned the youngest age group (18–24 years), and the domains pain/discomfort and anxiety/depression, where the absolute difference between the frequency of problems reported by women and men was 33.5% and 32.4%, respectively (Supplemental Fig. 1).

In total, we observed 170 different health states (out of the 3125 possible defined by EQ-5D-5L). The five most common health states accounted for 50.7% of cases, while the ten most common accounted for 60.8% of cases (Table 3). Two hundred seventy-nine respondents (27.4%) did not report any health restrictions (health state ‘11111’).

**Table 3:** Most common EQ-5D-5L health states among the Moscow population

Health state	*n*	%	Mean EQ-5D-5L Index	Mean EQ VAS			*n* males	*n* females
				All	Males	Females		
11111	279	27.4	1.000	84.9	85.2	84.6	154	125
11112	85	8.3	0.967	82.6	81.2	83.8	40	45
11121	55	5.4	0.947	77.7	77.5	78.0	27	28
11122	54	5.3	0.915	77.2	81.0	75.9	14	40
21121	44	4.3	0.913	72.5	73.5	71.8	20	24
21122	25	2.5	0.881	67.7	74.0*	63.5*	10	15
11222	21	2.1	0.856	67.0	69.4	65.3	9	12
21111	21	2.1	0.966	73.3	71.0	75.5	10	11
11211	19	1.9	0.941	78.3	80.0	76.4	10	9
11212	17	1.7	0.909	78.0	81.0	75.2	8	9
11123	16	1.6	0.906	70.5	72.6	68.9	7	9
11221	16	1.6	0.889	73.1	71.0	74.1	5	11
21222	16	1.6	0.822	71.2	76.3	69.5	4	12
11113	12	1.2	0.959	70.0	69.0	75.0	10	2
11213	12	1.2	0.900	70.8	63.3	73.3	3	9
21112	12	1.2	0.934	73.6	70.8	76.3	6	6
21221	12	1.2	0.922	67.3	63.7	70.8	6	6

*p < 0.05 males vs females, Mann–Whitney test

The whole sample distribution of the EQ-5D-5L index, EQ VAS and LSS is presented in Supplemental Fig. 2 (parallel chart created in IBM Watson Studio Cloud). As in other general population studies, the health-related quality of life of Moscow residents measured according to the EQ-5D-5L index or LSS, clustered around the highest values, whereas when measured by EQ VAS it was far from the top.

Detailed characteristics of the EQ-5D-5L index and EQ VAS, according to gender and age group, are shown in Table 4. Similar data summary concerning LSS is presented in Supplemental Table 1.

**Table 4:** EQ-5D-5L index and EQ VAS according to age group and gender

Age group (years)	All		Male		Female	
	*n*	Mean (SD) [95% CI]	*n*	Mean (SD) [95% CI]	*n*	Mean (SD) [95% CI]
EQ-5D-5L Index						
All ages	1020	0.907 (0.106) [0.900–0.913]	465	0.923 (0.083) [0.918–0.931]	555	0.893 (0.120) [0.883–0.903]
18–24	184	0.934 (0.090) [0.921–0.947]	85	0.960 (0.056) [0.948–0.972]	99	0.911 (0.106) [0.890–0.933]
25–34	190	0.937 (0.074) [0.927–0.948]	83	0.937 (0.070) [0.921–0.953]	107	0.937 (0.077) [0.922–0.952]
35–44	170	0.938 (0.064) [0.929–0.948]	76	0.952 (0.060) [0.938–0.966]	94	0.927 (0.065) [0.914–0.940]
45–54	150	0.917 (0.081) [0.904–0.930]	69	0.935 (0.075) [0.917–0.953]	81	0.902 (0.083) [0.884–0.921]
55–64	126	0.908 (0.084) [0.891–0.921]	59	0.928 (0.066) [0.911–0.945]	67	0.886 (0.094) [0.863–0.909]
65–74	106	0.855 (0.012) [0.834–0.878]	50	0.878 (0.079) [0.856–0.900]	56	0.835 (0.143) [0.797–0.873]
75+	94	0.778 (0.169) [0.744–0.813]	43	0.802 (0.099) [0.771–0.832]	51	0.759 (0.210) [0.700–0.818]
EQ VAS						
All ages	1020	74.1 (17.3) [73.0–75.1]	465	75.3 (17.4) [73.7–76.9]	555	73.0 (17.2) [71.6–74.5]
18–24	184	78.7 (14.8) [76.6–80.9]	85	83.2 (12.1) [80.6–85.8]	99	74.9 (15.9) [71.7–78.0]
25–34	190	80.2 (13.0) [78.3–82.0]	83	80.2 (13.9) [77.2–83.2]	107	80.2 (12.3) [77.8–79.0]
35–44	170	77.5 (14.4) [75.3–79.7]	76	79.1 (12.8) [76.1–82.0]	94	76.3 (15.6) [73.1–79.4]
45–54	150	75.3 (16.8) [72.6–78.0]	69	78.0 (16.9) [73.9–82.0]	81	73.1 (16.6) [69.4–76.7]
55–64	126	74.2 (15.2) [71.6–76.9]	59	73.2 (15.1) [69.3–77.2]	67	75.1 (15.4) [71.3–78.8]
65–74	106	62.2 (18.0) [58.7–65.6]	50	62.8 (17.7) [57.7–67.8]	56	61.6 (18.3) [56.7–66.5]
75+	94	57.4 (20.5) [53.2–61.6]	43	56.4 (21.9) [49.7–63.1]	51	58.3 (19.4) [52.9–63.8]

In general, lower HRQoL values start to be evident from the age of 45, and the decline in HRQoL accelerates from 65 years old. Women are characterised by worse HRQoL ratings than men, except for two age groups (55–64 years, 75+ years) in EQ VAS.

Electronic Supplementary Material (Supplemental Tables 2–9, Supplemental Figs. 3–8) presents the descriptive analysis of population data, preceding the regression analysis. Better educated respondents tend to report fewer limitations within UA, but more within AD. Young (18–24 years) males with primary or secondary education reported fewer problems within all EQ-5D-5L dimensions, than those with higher education. Young (18–24 years) females with primary or secondary education reported substantially fewer problems within PD and AD dimensions (by 14.7% and 22.1%, respectively), than those with higher education. Better educated women had higher EQ VAS and EQ-5D-5L index scores across all age groups. Relationship between current smoking and HRQoL was unsystematic. Higher income was associated with better EQ VAS and EQ-5D-5L index scores, fewer problems within MO, UA and PD dimensions, but more anxiety and depression.

Table 5 presents the results of multivariate analysis on socio-demographic characteristics, which significantly predicted HRQoL outcomes. Female sex, advanced age (65+ years) and lack of access to the Internet had a negative influence. In contrast, residence in selected districts (Zelenograd, North-West, South-East) had a positive impact on health status, as measured by the EQ-5D-5L index. The results for EQ VAS were similar, although not statistically significant for district-based dependences.

**Table 5:** Relation of EQ-5D-5L index and EQ VAS with demographic characteristics of respondents (*N* = 1020)

	*n* (%)	EQ Index		EQ VAS	
		Mean (SD)	Multiple linear regression coefficients	Mean (SD)	Multiple linear regression coefficients
Intercept			0.938		78.4
Gender					
Male	465 (45.6)	0.923 (0.083)	–	75.3 (17.4)	–
Female	555 (54.4)	0.893 (0.120)	− 0.034**	73.0 (17.2)	− 2.5**
Age group					
18–24 years	184 (18.0)	0.934 (0.090)	–	78.7 (14.8)	–
25–44 years	360 (35.3)	0.938 (0.069)	− 0.001	78.9 (13.7)	− 0.7
45–64 years	276 (27.1)	0.912 (0.082)	− 0.016	74.8 (16.1)	− 3.3**
65+ years	200 (19.6)	0.819 (0.149)	− 0.092**	60.0 (19.3)	− 16.7**
Education level					
Secondary or primary	417 (41.0)	0.901 (0.121)	–	73.2 (19.2)	–
Higher	599 (59.0)	0.911 (0.094)	0.011	74.6 (15.9)	2.0*
Smoking status					
No	736 (72.7)	0.904 (0.108)	–	73.2 (17.0)	–
Yes	276 (27.3)	0.914 (0.101)	− 0.007	76.2 (18.1)	0.9
Internet usage					
Everyday	785 (77.9)	0.927 (0.079)	–	77.1 (14.8)	–
Minimum once a week	81 (8.0)	0.883 (0.096)	− 0.011	66.3 (18.7)	− 5.0**
No	142 (14.1)	0.811 (0.170)	− 0.059**	62.4 (22.6)	− 4.3**
District					
North-East	118 (11.6)	0.890 (0.107)	–	72.3 (17.9)	–
Central	69 (6.8)	0.903 (0.118)	0.006	74.1 (15.5)	0.7
East	126 (12.4)	0.896 (0.106)	0.005	73.5 (19.9)	0.8
North	98 (9.6)	0.912 (0.114)	0.020	75.5 (15.2)	2.5
North-West	88 (8.6)	0.920 (0.087)	0.029**	76.0 (16.7)	2.8
South	148 (14.5)	0.903 (0.131)	0.010	72.4 (16.8)	− 0.7
South-East	116 (11.4)	0.916 (0.093)	0.026**	73.1 (19.0)	0.3
South-West	121 (11.9)	0.913 (0.086)	0.020	75.6 (15.2)	2.5
West	116 (11.4)	0.908 (0.107)	0.012	74.5 (18.7)	0.9
Zelenograd	20 (2.0)	0.940 (0.060)	0.044*	78.5 (11.7)	5.3

*p < 0.1; **p < 0.05

## Discussion

To the best of our knowledge, this is the first study to estimate population norms for the descriptive part of the EQ-5D-5L questionnaire, EQ VAS, EQ-5D-5L index and Level Sum Score, among a representative sample of Moscow inhabitants. Norms for the EQ-5D-5L index were obtained through the usage of a recently developed Russian EQ-5D-3L value set and EuroQol Group cross-over methodology. Moscow’s normative data may be used as reference values in future EQ-5D-based studies. We have found that HRQoL is worse in women than in men across almost all age groups, especially in the youngest one (18–24 years).

One of the limitations of our study may be the moderate sample size (about 1000 respondents), which is clearly smaller than, for example, that used in a Spanish study (> 20,000 of respondents) [14, 15]. We would like to point out, however, that the sample size should be correlated with the size of the target population. If we estimate the number of respondents per 1 million of the population (about 86 in the case of our study), this indicator is higher than in most of the EQ-5D-5L population norms studies which we identified—in Germany [16–18], Ireland [19], Uruguay [20], South Korea [21], Japan [22], the USA [23] and Indonesia [24].

Although since July 2012 there has existed a new official administrative division of Moscow (two new districts were added), we decided to limit our study to Moscow’s former division into 10 zones. The two new areas have a suburban character, are distant from the city centre (up to 80 km), less populated, have limited access to the metro system, and the health-related quality of life of people living there could be different than for other Muscovites.

Our sample proved to be representative for the Moscow population in terms of sex, age group and the district of residence. However, we based our study on quota sampling, which is a less optimal solution than the random sampling used in other Central and Eastern European studies, such as in Slovenia or Poland (computer selection of respondents based on phone number or personal identification number) [25, 26].

Although accessibility of the Moscow transportation system for the disabled has improved over the last few years, since the Passenger Mobility Centre was opened in 2013, limitations for handicapped people on Moscow transportation systems remains significant, and we were not able to interview any person with a visible disability. According to official statistics, disabled people constitute about 8.5% of the Russian population [27]. One may wonder whether the results of our study would be different if it had taken the form of a postal survey or a direct interview at the respondent’s home.

Our sample was not perfectly balanced in terms of education. Higher education constitutes 42% in Moscow reference data and 59% in our study group, primary education—6.5% and 0.5%, respectively. In general, the level of education of Muscovites seems high compared to other populations. Our sample was additionally skewed in the direction of higher levels of education. This could be a particular limitation in situations in which Moscow population norms will be used to evaluate health care programmes that affect the less-educated set of beneficiaries, for example, citizens of other Russian regions.

A high percentage of respondents (about 22%) refused to answer the question regarding income. As stated previously, refusal to answer among Russian respondents, particularly in Moscow, is frequently of significant proportions, partly due to high levels of distrust towards strangers [28].

There is a long tradition of health surveys carried out among the Moscow population. In the last 3 decades several have taken place: a postal survey in 1991 (*n* = 545) [29], the Moscow Health Survey in 2004 (*n* = 1190) [18, 30, 31] or the Stress, Ageing, and Health in Russia (SAHR) study in 2006–2011 (*n* = 1800) [32, 33]. In the first two of these, health-related quality of life measurement was based on a single-item self-reported measure; in the last one, it was done using the 36-item Short Form Health Survey (SF-36).

We did not identify any population norms survey, neither in Moscow nor Russia, based on the EQ-5D-5L questionnaire. However, in general, studies including EQ-5D are quite numerous, especially those undertaken in disease-specific populations and published in the Russian language. The Russian version of EQ-5D-5L has been successfully validated in a group of 163 patients with spondylarthritis [34]. Recent years have witnessed attempts to carry out EQ-5D-3L valuation [35, 36], which should soon result in the final publication of a country-specific Russian value set [11]. The availability of validated tools should facilitate international comparisons, which up to now have been based mainly on general economic and social indicators [37–39].

The lower health perceptions among Moscow women was noted in the early nineties in a study comparing them with women from Helsinki [19], and subsequently in a comparison with Danish citizens [40] and in the Moscow Health Study [18, 21]. Similar results were found in a study of women from St. Petersburg, Russia’s second-largest city [41]. Self-rated health was much worse in St. Petersburg than in Estonia or Finland. Housewives, in comparison to employed women, had better self-rated health, unlike in the two other areas [32]. Studies indicate that in Russia, although females outlive men and the difference in life expectancy is one of the world’s most significant [42], they generally report worse health [43, 44]. This phenomenon, known as the male–female health-survival paradox [45], is very pronounced in Moscow [31].

One of the potentially interesting results of our study is the evidence of a particularly low HRQoL in young females, as measured with standardised tools. Among women aged 18–24 years, over 60% reported problems with anxiety/depression and over 53% with pain/discomfort. The assessment of health with EQ VAS was 8.3 points lower than in men from the same age group.

Many different factors could lie behind this phenomenon. The Russian sociologist Elena Varshavskaya [46] writes about the NEET group (Not in Employment, Education or Training) among Russian youth. In 2010, Eurostat adopted the standardised definition of NEET and developed a methodology for the statistical calculation of its level. The NEET group includes young people aged 15–24 years who are unemployed or economically inactive, and at the same time do not study and are not covered by vocational training. According to Varshavskaya [46], the proportion of those who do not work or study is about 5% higher among women than among men (15.5 and 10.4%, respectively). Engagement in fulfilling activities, such as education, training, volunteering or work, contributes to future financial independence and is essential for well-being [47].

HRQoL population norms for Moscow, taking into account the size of the city—over 11.8 million inhabitants, constitute a standalone value. Nevertheless, our study may be treated as a pilot project, preceding a nation-wide survey. Given the geographical extent of the country, the implementation of Russia population norms study can be a challenge but should constitute the next logical stage of research. Current Moscow population norms should be re-estimated, when the directly measured EQ-5D-5L, becomes available.

## Conclusions

This study presents population norms for the EQ-5D-5L health questionnaire, based on a representative sample of Moscow inhabitants. These reference values, distinguishing age and gender groups, can be used to optimise the effectiveness of resource allocation in healthcare.

## Electronic supplementary material

Below is the link to the electronic supplementary material.

Supplementary file1 (PDF 424 KB)

## Data Availability

No data were collected that could identify the respondent.

## Data availability

The datasets generated during the current study are available from the corresponding author on reasonable request.

## References

[1] Rencz F, Gulácsi L, Drummond M, Golicki D, Prevolnik Rupel V, Simon J, Stolk EA, Brodszky V, Baji P, Závada J, Petrova G, Rotar A, Péntek M (2016). EQ-5D in Central and Eastern Europe: 2000–2015. Quality of Life Research.

[2] Holownia-Voloskova M, Vorobiev PA, Grinin M, Davydovskaya MV, Ermolaeva TN, Kokushkin KA (2018). Drug policy in the Russian Federation. Value in Health Regional Issues.

[3] Fayers PM, Hays R (2005). Assessing quality of life in clinical trials.

[4] EuroQol Research Foundation: EQ-5D is a recommended tool for use in cost-utility analyses around the globe. https://euroqol.org/eq-5d-is-a-recommended-tool-for-use-in-cost-utility-analyses-around-the-globe/ (2019). Accessed 24 March 2020.

[5] Brooks R (1996). EuroQol: The current state of play. Health Policy.

[6] Herdman M, Gudex C, Lloyd A, Janssen M, Kind P, Parkin D, Bonsel G, Badia X (2011). Development and preliminary testing of the new five-level version of EQ-5D (EQ-5D-5L). Quality of Life Research.

[7] Janssen MF, Pickard AS, Golicki D, Gudex C, Niewada M, Scalone L, Swinburn P, Busschbach J (2013). Measurement properties of the EQ-5D-5L compared to the EQ-5D-3L across eight patient groups: A multi-country study. Quality of Life Research.

[8] Szende A, Janssen B, Cabases J (2014). Self-reported population health: An international perspective based on EQ-5D.

[9] Deyev AI (2018). How do Muscovites live longer?. Uspekhi gerontologii.

[10] ROSSTAT, Federal State Statistic Service.: Population of the Russian Federation by municipalities as for-1.01.2014. http://www.gks.ru/wps/wcm/connect/rosstat_main/rosstat/ru/statistics/publications/catalog/afc8ea004d56a39ab251f2bafc3a6fce (2014). Accessed 30 June 2019.

[11] Golicki D, Jakubczyk M, Graczyk K, Niewada M (2019). Valuation of EQ-5D-5L health states in Poland: The first EQ-VT-based study in Central and Eastern Europe. Pharmacoeconomics.

[12] Omelyanovskiy, V., Musina, N. Z., Ratushnyak, S. S., Bezdenezhnyh, T. P., Fediaeva, V. K., Roudijk, B., Purba, F. D. (2020). Preliminary results of the EQ-5D-3L valuation study in Russia. 4th EuroQol Academy Meeting, Prague, Czech Republic, 2–4 March.

[13] van Hout B, Janssen MF, Feng YS, Kohlmann T, Busschbach J, Golicki D, Lloyd A, Scalone L, Kind P, Pickard AS (2012). Interim scoring for the EQ-5D-5L: Mapping the EQ-5D-5L to EQ-5D-3L value sets. Value in Health.

[14] Garcia-Gordillo MA, Adsuar JC, Olivares PR (2016). Normative values of EQ-5D-5L: In a Spanish representative population sample from Spanish Health Survey, 2011. Quality of Life Research.

[15] Hernandez G, Garin O, Pardo Y, Vilagut G, Pont A, Suarez M, Neira M, Rajmil L, Gorostiza I, Ramallo-Farina Y, Cabases J, Alonso J, Ferrer M (2018). Validity of the EQ-5D-5L and reference norms for the Spanish population. Quality of Life Research.

[16] Hinz A, Kohlmann T, Stobel-Richter Y, Zenger M, Brahler E (2014). The quality of life questionnaire EQ-5D-5L: Psychometric properties and normative values for the general German population. Quality of Life Research.

[17] Huber MB, Felix J, Vogelmann M, Leidl R (2017). Health-related quality of life of the general German population in 2015: Results from the EQ-5D-5L. International Journal of Environmental Research and Public Health.

[18] Grochtdreis T, Dams J, Konig HH, Konnopka A (2019). Health-related quality of life measured with the EQ-5D-5L: Estimation of normative index values based on a representative German population sample and value set. The European Journal of Health Economics.

[19] Hobbins A, Barry L, Kelleher D, O’Neill C (2018). The health of the residents of Ireland: Population norms for Ireland based on the EQ-5D-5L descriptive system—a cross sectional study. HRB Open Research.

[20] Augustovski F, Rey-Ares L, Irazola V, Garay OU, Gianneo O, Fernandez G, Morales M, Gibbons L, Ramos-Goni JM (2016). An EQ-5D-5L value set based on Uruguayan population preferences. Quality of Life Research.

[21] Kim TH, Jo MW, Lee S, Kim SH, Chung SM (2013). Psychometric properties of the EQ-5D-5L in the general population of South Korea. Quality of Life Research.

[22] Shiroiwa T, Fukuda T, Ikeda S, Igarashi A, Noto S, Saito S, Shimozuma K (2016). Japanese population norms for preference-based measures: EQ-5D-3L, EQ-5D-5L, and SF-6D. Quality of Life Research.

[23] Craig BM, Pickard AS, Lubetkin EI (2014). Health problems are more common, but less severe when measured using newer EQ-5D versions. Journal of Clinical Epidemiology.

[24] Purba FD, Hunfeld JAM, Iskandarsyah A, Fitriana TS, Sadarjoen SS, Passchier J, Busschbach JJV (2018). Quality of life of the Indonesian general population: Test-retest reliability and population norms of the EQ-5D-5L and WHOQOL-BREF. PLoS One.

[25] Klemenc-Ketis Z, Smogavec M, Softic N, Kersnik J (2011). Health-related quality of life: A population based study from Slovenia. Central European Journal of Public Health.

[26] Golicki D, Niewada M (2017). EQ-5D-5L Polish population norms. Archives of Medical Science.

[27] Data of Federal Statistic Bureau of Russian Federation. http://www.gks.ru/wps/wcm/connect/rosstat_main/rosstat/ru/statistics/population/disabilities/#. Accessed 30 June 2019.

[28] Vågerö D, Kislitsyna O, Ferlander S, Migranova L, Carlson P, Rimachevskaya N (2008). Moscow Health Survey 2004—Social surveying under difficult circumstances. International Journal of Public Health.

[29] Palosuo H, Uutela A, Zhuravleva I, Lakomova N (1998). Social patterning of ill health in Helsinki and Moscow: Results from a comparative survey in 1991. Social Science and Medicine.

[30] Ferlander S, Mäkinen IH (2009). Social capital, gender and self-rated health. Evidence from the Moscow Health Survey 2004. Social Science & Medicine.

[31] Ferlander S, Stickley A, Kislitsyna O, Jukkala T, Carlson P, Mäkinen IH (2016). Social capital—A mixed blessing for women? A cross-sectional study of different forms of social relations and self-rated depression in Moscow. BMC Psychology.

[32] Shkolnikova M, Shalnova S, Shkolnikov VM, Metelskaya V, Deev A, Andreev E, Jdanov D, Vaupel JW (2009). Biological mechanisms of disease and death in Moscow: Rationale and design of the survey on Stress Aging and Health in Russia (SAHR). BMC Public Health.

[33] Oksuzyan A, Shkolnikova M, Vaupel JW, Christensen K, Shkolnikov VM (2015). Sex differences in biological markers of health in the study of stress, aging and health in Russia. PLoS One.

[34] Akulova AI, Gaydukova IZ, Rebrov AP (2018). Validation of the EQ-5D-5L version in Russia. Nauchno-Prakticheskaya Revmatologiya.

[35] Kind P, Gerry C (2017). From Russia with love—Valuation of EQ-5D health states using available data. Value in Health.

[36] Khabibullina A, Gerry CJ (2019). Valuing health states in Russia: A first feasibility study. Value in Health Regional Issues.

[37] Liu Y, Rao K, Fei J (1998). Economic transition and health transition: Comparing China and Russia. Health Policy.

[38] Jogerst GJ, Daly JM, Hesli V, Saha C (2006). Comparison of health and effective functioning in Russia and the United States. Clinical Interventions in Aging.

[39] Hsieh N (2015). Economic security, social cohesion, and depression disparities in post-transition societies: A comparison of older adults in China and Russia. Journal of Health and Social Behavior.

[40] Oksuzyan A, Shkolnikova M, Vaupel JW, Christensen K, Shkolnikov VM (2014). Sex differences in health and mortality in Moscow and Denmark. European Journal of Epidemiology.

[41] Dubikaytis T, Härkänen T, Regushevskaya E, Hemminki E, Haavio-Mannila E, Laanpere M, Kuznetsova O, Koskinen S (2014). Socioeconomic differences in self-rated health among women: A comparison of St. Petersburg to Estonia and Finland. International Journal for Equity in Health.

[42] Field M, Shkolnikov V, Andreev E, Maleva T (2000). Gender gaps in mortality. Dissimilarities in mortality rates: Analysis of standard data. Inequality and mortality in Russia.

[43] Rose R (2000). How much does social capital add to individual health? A survey study of Russians. Social Science and Medicine.

[44] Gordeev VS, Goryakin Y, McKee M, Stuckler D, Roberts B (2016). Economic shocks and health resilience: Lessons from the Russian Federation. Journal of Public Health.

[45] Nathanson CA (1975). Illness and the feminine role: A theoretical review. Social Science and Medicine.

[46] Varshavskaya EA (2016). Rossiyskaya NEET-molodezh': kharakteristiki i tipologiya. Sotsiologicheskiye issledovaniya.

[47] Office for National Statistics.: Young people’s well-being. https://www.ons.gov.uk/peoplepopulationandcommunity/wellbeing/articles/youngpeopleswellbeingandpersonalfinance/2017 (2017). Accessed 30 May 2020.

