# Supplementary material for: Population norms of health-related quality of life in Moscow, Russia: the EQ-5D-5L-based survey
Source: Qual Life Res. 2020 Nov 25;30(3):831–40. doi: 10.1007/s11136-020-02705-0 (PMC7952340; doi:10.1007/s11136-020-02705-0)

Electronic Supplementary Material

Title: Population norms of health-related quality of life in Moscow, Russia: the EQ-5D-5L-based survey

Journal: Quality of Life Research

Authors

Malwina Hołownia-Voloskova, Alexey Tarbastaev, Dominik Golicki

Table of Contents:

Supplemental Fig. 1 Prevalence of health limitations (any level of severity) for EQ-5D-5L dimensions according to age group and sex: (A) males, (B) females........ 2

Supplemental Fig. 2 Distribution of (A) EQ-5D-5L index, (B) EQ VAS scores and (C) Level Sum Score among the Moscow population 4

Supplemental Table 1 Level Sum Score (LSS) according to age group and gender........ 5

Supplemental Table 2 Any problems (%) within EQ-5D-5L dimensions according to level of education (secondary or primary, higher) and age group: both sexes 6

Supplemental Table 3 Any problems (%) within EQ-5D-5L dimensions according to level of education (secondary or primary, higher) and age group: males........ 7

Supplemental Table 4 Any problems (%) within EQ-5D-5L dimensions according to level of education (secondary or primary, higher) and age group: females 8

Supplemental Table 5 EQ VAS according to level of education (secondary or primary, higher), age group and sex........ 9

Supplemental Table 6 EQ-Index according to level of education (secondary or primary, higher), age group and sex........ 10

Supplemental Table 7 Any problems (%) within EQ-5D-5L dimensions according to smoking status (current smoker, no smoking) and age group. 11

Supplemental Table 8 EQ VAS and EQ-Index according to smoking status (current smoker, no smoking) and age group. 12

Supplemental Table 9 Any problems (%) within EQ-5D-5L dimensions according to income group. 13

Supplemental Fig. 3 Any problems (%) within EQ-5D-5L dimensions according to monthly income group........ 13

Supplemental Fig. 4 EQ VAS according to monthly income group. 14

Supplemental Fig. 5 EQ-Index according to monthly income group. 14

Supplemental Fig. 6 Any problems (%) within EQ-5D-5L dimensions according to Internet usage. .. 15

Supplemental Fig. 7 EQ VAS according to Internet usage 15

Supplemental Fig. 8 EQ-Index according to Internet usage. 16

Supplemental Fig. 1 Prevalence of health limitations (any level of severity) for EQ-5D-5L dimensions according to age group and sex: (A) males, (B) females

A

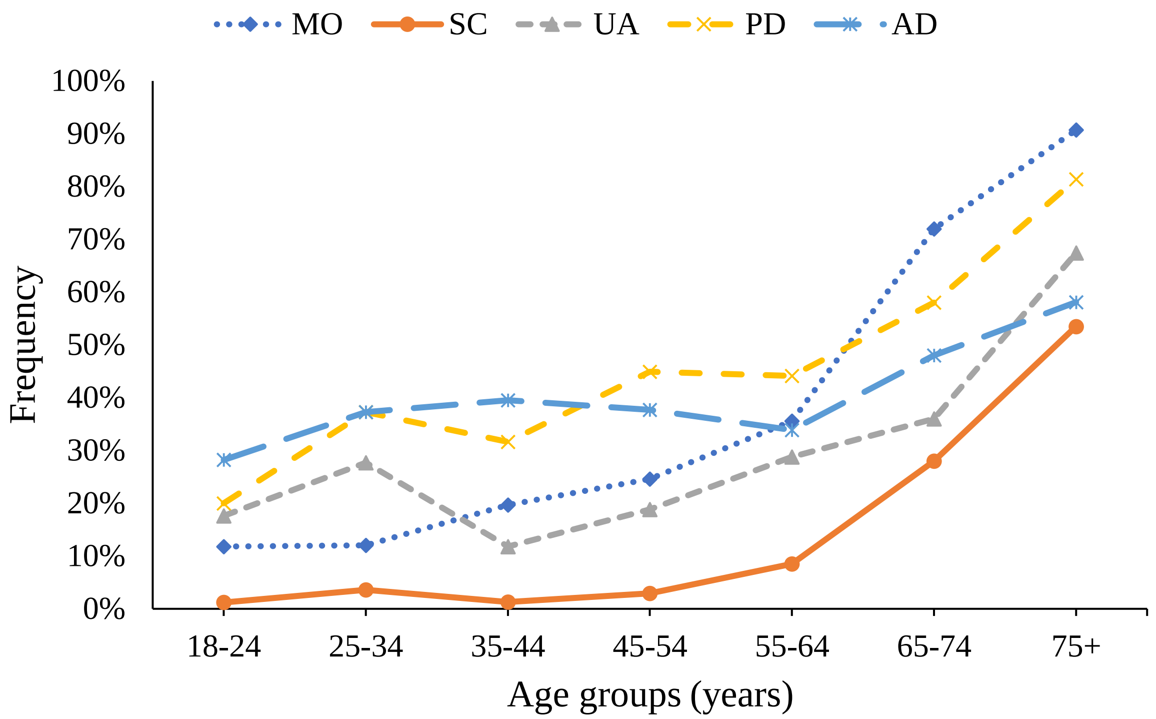

B

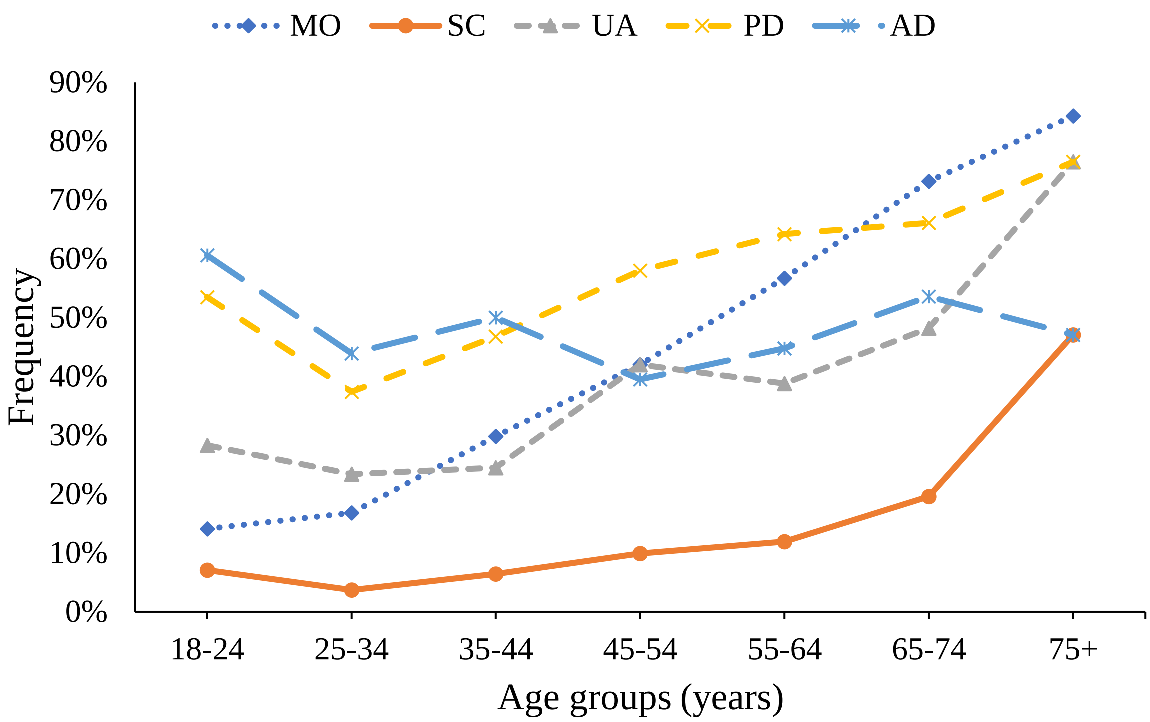

MO
SC
UA
PD
AD
90%
80%
70%
60%
50%
40%
30%
20%
10%
0%
Frequency
18-24
25-34
35-44
45-54
55-64
65-74
75+
Age groups (years)

Supplemental Fig. 2 Distribution of (A) EQ-5D-5L index, (B) EQ VAS scores and (C) Level Sum Score among the Moscow population

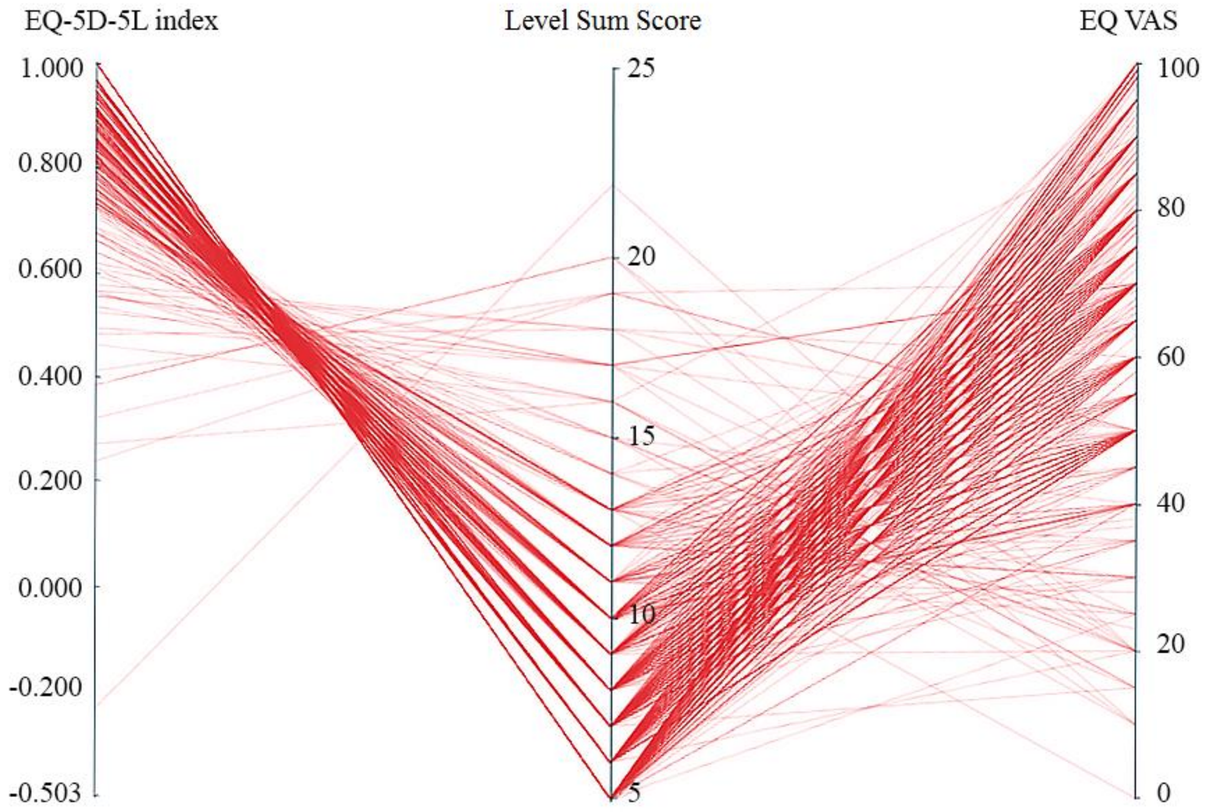

Supplemental Table 1 Level Sum Score (LSS) according to age group and gender

Age group (years)	All		Male		Female	
	n	Mean (SD) [95%CI]	n	Mean (SD) [95%CI]	n	Mean (SD) [95%CI]
All ages	1020	7.3 (2.6) [7.2–7.5]	465	7.0 (2.2) [6.7–7.2]	555	7.7 (2.8) [7.4-7.9]
18-24	184	6.5 (1.9) [6.3–6.8]	85	6.0 (1.4) [5.7–6.3]	99	7.0 (2.1) [6.6–7.4]
25-34	190	6.6 (1.9) [6.3–6.9]	83	6.6 (1.8) [6.2–7.0]	107	6.6 (2.0) [6.2-7.0]
35-44	170	6.6 (1.6) [6.3–6.8]	76	6.3 (1.5) [5.9–6.6]	94	6.8 (1.7) [6.5–7.2]
45-54	150	7.1 (2.1) [6.7–7.4]	69	6.6 (2.0) [6.2–7.1]	81	7.4 (2.1) [7.0–7.9]
55-64	126	7.4 (2.1) [7.0–7.8]	59	6.8 (1.6) [6.4–7.2]	67	7.9 (2.3) [7.3–7.6]
65-74	106	8.7 (2.9) [8.2–9.3]	50	8.1 (2.1) [7.5–8.7]	56	9.3 (3.5) [8.3–10.2]
75+	94	10.7 (3.6) [9.9–11.4]	43	10.2 (2.8) [9.3–11.0]	51	11.0 (4.2) [9.9–12.2]

Supplemental Table 2 Any problems (%) within EQ-5D-5L dimensions according to the level of education (secondary or primary, higher) and age group: both sexes

Both sexes	All ages	Age group			
		18-24 years	25-44 years	45-64 years	65+ years
Secondary or primary education					
MO	37.2	12.5	29.3	42.3	89.7
SC	11.3	14.2	8.5	9.8	32.4
UA	33.8	23.6	26.8	33.3	64.7
PD	49.9	44.0	45.1	53.7	82.4
AD	42.2	40.3	39.0	37.4	58.8
Higher education					
MO	34.9	15.0	17.0	37.9	75.4
SC	11.7	5.0	2.5	7.2	38.5
UA	30.6	12.5	20.7	32.0	52.3
PD	47.9	52.5	37.0	52.9	63.8
AD	45.4	65.0	44.2	40.5	47.7
Difference (secondary/primary education - higher education)*					
MO	2.3	-2.5	12.3	4.4	14.3
SC	-0.4	9.2	6.0	2.6	-6.1
UA	3.2	11.1	6.1	1.3	12.4
PD	2.0	-8.5	8.1	0.8	18.6
AD	-3.2	-24.7	-5.2	-3.1	11.1

* Green colour means that respondents with higher education have less problems than respondents with secondary/primary education. Red colour means the opposite - respondents with higher education have more problems than respondents with secondary/primary education.

Supplemental Table 3 Any problems (%) within EQ-5D-5L dimensions according to the level of education (secondary or primary, higher) and age group: males

Males	All ages	Age group			
		18-24 years	25-44 years	45-64 years	65+ years
Secondary or primary education					
MO	31.3	11.3	29.5	30.2	88.9
SC	7.3	0.0	2.3	6.3	37.0
UA	26.3	16.9	25.0	23.8	59.3
PD	40.0	18.3	45.5	41.3	85.2
AD	33.2	25.4	31.8	33.3	55.6
Higher education					
MO	32.4	14.3	10.4	29.2	78.5
SC	13.1	7.1	2.6	4.6	41.5
UA	17.0	21.4	18.3	23.1	47.7
PD	42.5	28.6	30.4	47.7	61.5
AD	43.2	42.9	40.9	38.5	52.3
Difference (secondary/primary - higher education)*					
MO	-1.1	-3.0	19.1	1.0	10.4
SC	-5.8	-7.1	-0.3	1.7	-4.5
UA	9.3	-4.5	6.7	0.7	11.6
PD	-2.5	-10.3	15.1	-6.4	23.7
AD	-10.0	-17.5	-9.1	-5.2	3.3

* Green colour means that respondents with higher education have less problems than respondents with secondary/primary education. Red colour means the opposite - respondents with higher education have more problems than respondents with secondary/primary education.

Supplemental Table 4 Any problems (%) within EQ-5D-5L dimensions according to the level of education (secondary or primary, higher) and age group: females

Females	All ages	Age group			
		18-24 years	25-44 years	45-64 years	65+ years
Secondary or primary education					
MO	42.9	13.7	28.9	55.0	90.2
SC	15.1	8.2	15.8	13.3	29.3
UA	41.0	30.1	28.9	43.3	68.3
PD	59.4	50.7	44.7	66.6	80.5
AD	50.9	54.8	47.4	41.7	61.0
Higher education					
MO	36.8	15.4	21.7	44.3	72.3
SC	10.6	3.8	2.5	9.1	35.4
UA	33.3	23.1	2.4	38.6	56.9
PD	52.1	65.4	41.6	43.2	66.2
AD	47.1	76.9	46.6	42.0	43.1
Difference (secondary/primary - higher education)					
MO	6.1	-1.7	7.2	10.7	17.9
SC	4.5	4.4	13.3	4.2	-6.1
UA	7.7	7.0	26.5	4.7	11.4
PD	7.3	-14.7	3.1	23.4	14.3
AD	3.8	-22.1	0.8	-0.3	17.9

* Green colour means that respondents with higher education have less problems than respondents with secondary/primary education. Red colour means the opposite - respondents with higher education have more problems than respondents with secondary/primary education.

Supplemental Table 5 EQ VAS according to the level of education (secondary or primary, higher), age group and sex

	All ages	Age group			
		18-24 years	25-44 years	45-64 years	65+ years
Both sexes					
Secondary or primary education	73.2 (19.2)	78.4 (15.8)	76.3 (16.6)	74.0 (18.9)	56.8 (20.7)
Higher education	74.6 (15.9)	79.8 (10.7)	79.7 (12.7)	75.5 (13.5)	61.4 (18.4)
Difference (higher – secondary/primary)*	1.4	1.4	3.4	1.5	4.6
Males					
Secondary or primary education	76.2 (18.5)	83.1 (12.4)	76.3 (16.7)	76.8 (18.7)	56.3 (21.3)
Higher education	74.6 (16.5)	83.9 (11.0)	80.9 (11.6)	74.8 (13.5)	61.0 (19.3)
Difference (higher – secondary/primary)*	-1.6	0.8	4.6	-2.0	4.7
Females					
Secondary or primary education	70.3 (19.4)	73.9 (17.5)	76.3 (16.8)	71.1 (18.8)	57.1 (20.6)
Higher education	74.7 (15.5)	77.6 (10.1)	78.8 (13.4)	76.0 (13.6)	61.8 (17.7)
Difference (higher – secondary/primary)*	4.4	3.7	2.5	4.9	4.7

* Green colour means that respondents with higher education have higher EQ VAS values than respondents with secondary/primary education.
Red colour means the opposite - respondents with higher education have lower EQ VAS values than respondents with secondary/primary education.

Supplemental Table 6 EQ-5D-5L index according to the level of education (secondary or primary, higher), age group and sex

	All ages	Age group			
		18-24 years	25-44 years	45-64 years	65+ years
Both sexes					
Secondary or primary education	0.901 (0.121)	0.936 (0.096)	0.925 (0.080)	0.907 (0.097)	0.784 (0.171)
Higher education	0.911 (0.094)	0.925 (0.061)	0.942 (0.065)	0.916 (0.068)	0.837 (0.135)
Difference (higher – secondary/primary)*	0.010	-0.011	0.017	0.009	0.053
Males					
Secondary or primary education	0.927 (0.085)	0.963 (0.057)	0.932 (0.072)	0.930 (0.077)	0.821 (0.102)
Higher education	0.921 (0.082)	0.949 (0.049)	0.949 (0.063)	0.933 (0.063)	0.852 (0.094)
Difference (higher – secondary/primary)*	-0.006	-0.014	0.017	0.003	0.031
Females					
Secondary or primary education	0.875 (0.143)	0.911 (0.118)	0.916 (0.088)	0.883 (0.109)	0.760 (0.202)
Higher education	0.904 (0.103)	0.913 (0.064)	0.937 (0.065)	0.903 (0.069)	0.822 (0.165)
Difference (higher – secondary/primary)*	0.029	0.002	0.021	0.020	0.062

* Green colour means that respondents with higher education have higher EQ-5D-5L index values than respondents with secondary/primary education.
Red colour means the opposite - respondents with higher education have lower EQ-5D-5L index values than respondents with secondary/primary education.

Supplemental Table 7 Any problems (%) within EQ-5D-5L dimensions according to smoking status (current smoker, no smoking) and age group

	All ages	Age group			
		18-24 years	25-44 years	45-64 years	65+ years
Current smoker					
MO	30.1	18.5	21.9	29.1	86.2
SC	10.1	1.9	5.3	12.7	37.9
UA	31.9	37.0	26.3	31.6	44.8
PD	42.0	35.2	36.0	43.0	75.9
AD	41.3	50.0	38.6	34.2	55.2
No smoking					
MO	38.0	10.9	18.9	44.2	79.6
SC	12.1	15.5	3.3	6.6	36.5
UA	32.1	18.0	20.1	33.0	59.3
PD	51.5	39.8	40.2	57.4	70.1
AD	45.4	44.5	45.1	41.1	51.5
Difference (current smoker - no smoking)*					
MO	-7.9	7.6	3.0	-15.1	6.6
SC	-2.0	-13.6	2.0	6.1	1.4
UA	-0.2	19.0	6.2	-1.4	-14.5
PD	-9.5	-4.6	-4.2	-14.4	5.8
AD	-4.1	5.5	-6.5	-6.9	3.7

* Green colour means that non-smoking respondents have less problems than current smokers.
Red colour means the opposite - non-smoking respondents have more problems than current smokers.

Supplemental Table 8 EQ VAS and EQ-5D-5L index according to smoking status (current smoker, no smoking) and age group

	All ages	Age group			
		18-24 years	25-44 years	45-64 years	65+ years
EQ VAS, mean (SD)					
Current smoker	76.2 (18.1)	78.6 (16.5)	79.2 (15.5)	78.4 (15.2)	53.6 (22.0)
Non-smoker	73.2 (17.0)	78.7 (14.2)	78.8 (12.9)	73.4 (16.2)	60.8 (18.8)
Difference (non-smoker - current smoker)*	-3.0	0.1	-0.4	-5.0	7.2
EQ-5D-5L index, mean (SD)					
Current smoker	0.914 (0.101)	0.911 (0.127)	0.934 (0.077)	0.919 (0.092)	0.828 (0.113)
Non-smoker	0.904 (0.108)	0.943 (0.067)	0.940 (0.064)	0.909 (0.078)	0.815 (0.156)
Difference (non-smoker - current smoker)*	-0.010	0.032	0.006	-0.010	-0.013

* Green colour means that non-smokers have higher EQ VAS / EQ-5D-5L index values than current smokers.
Red colour means the opposite - non-smokers have lower EQ VAS / EQ-5D-5L index values than current smokers.

Supplemental Table 9 Any problems (%) within EQ-5D-5L dimensions according to monthly income group

EQ-5D-5L dimension	Monthly income level (RUB)					
	≤15,000	15,001-30,000	30,001-50,000	50,001-80,000	>80,000	Refused to answer
MO	39.2	53.7	36.2	32.0	17.6	27.3
SC	16.7	22.4	5.7	4.1	6.1	9.7
UA	32.4	44.3	39.1	29.9	14.5	23.6
PD	47.1	59.8	52.3	44.9	38.2	43.1
AD	50.0	49.2	43.1	36.7	50.4	37.5

Supplemental Fig. 3 Any problems (%) within EQ-5D-5L dimensions according to monthly income group

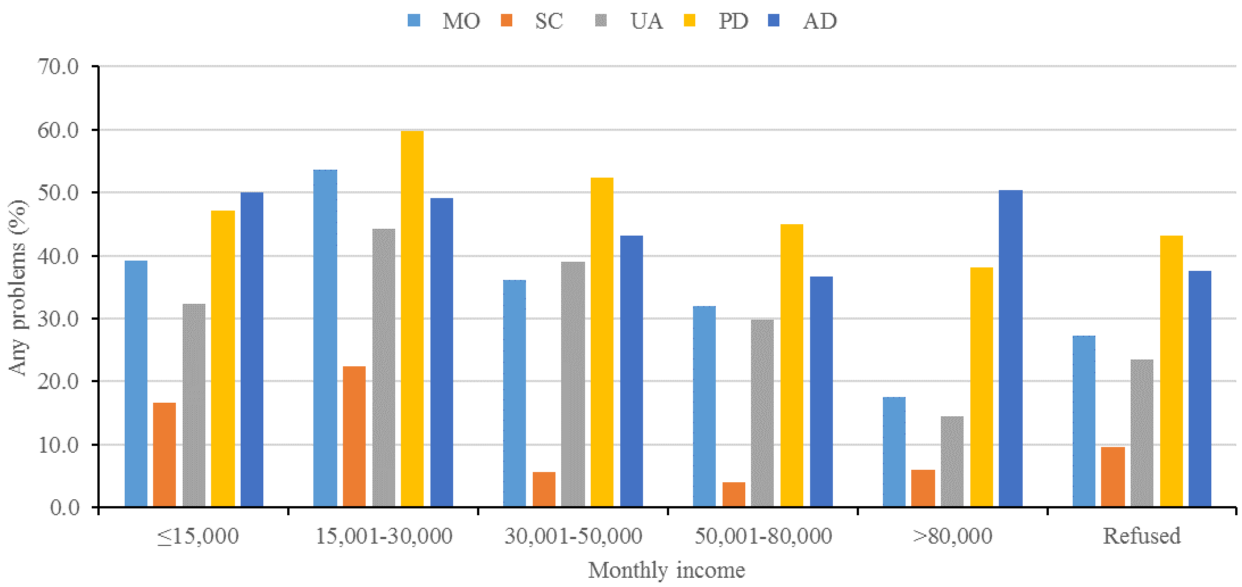

Supplemental Fig. 4 EQ VAS according to monthly income group

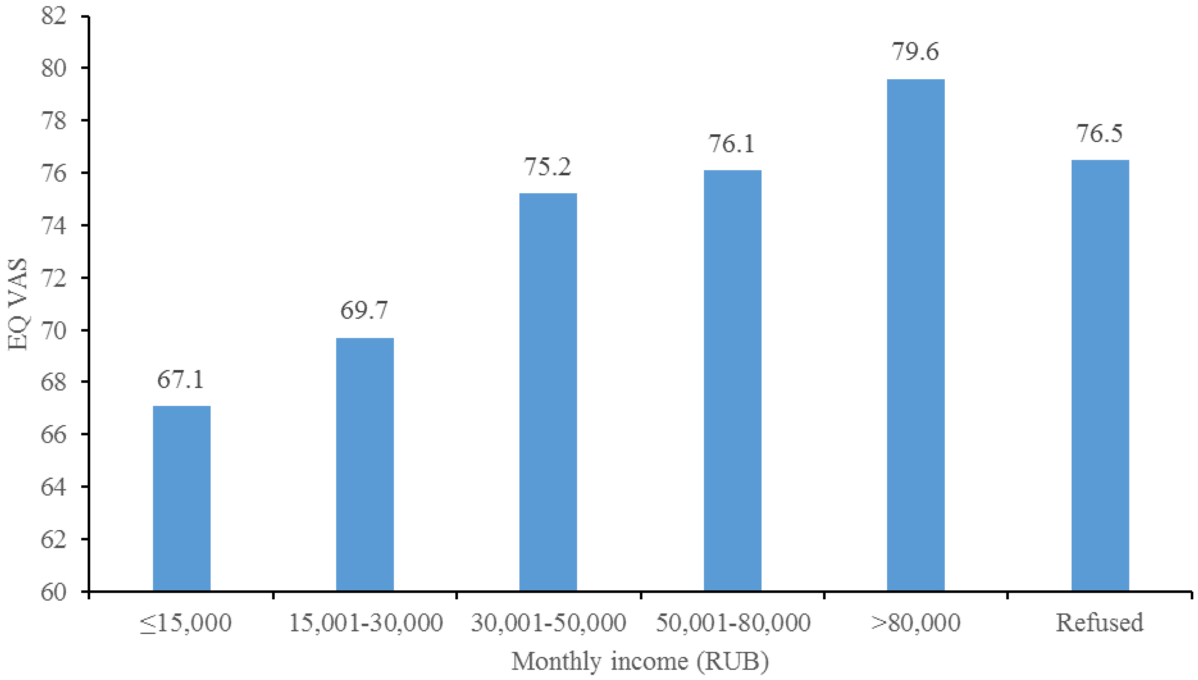

Supplemental Fig. 5 EQ-5D-5L index according to monthly income group

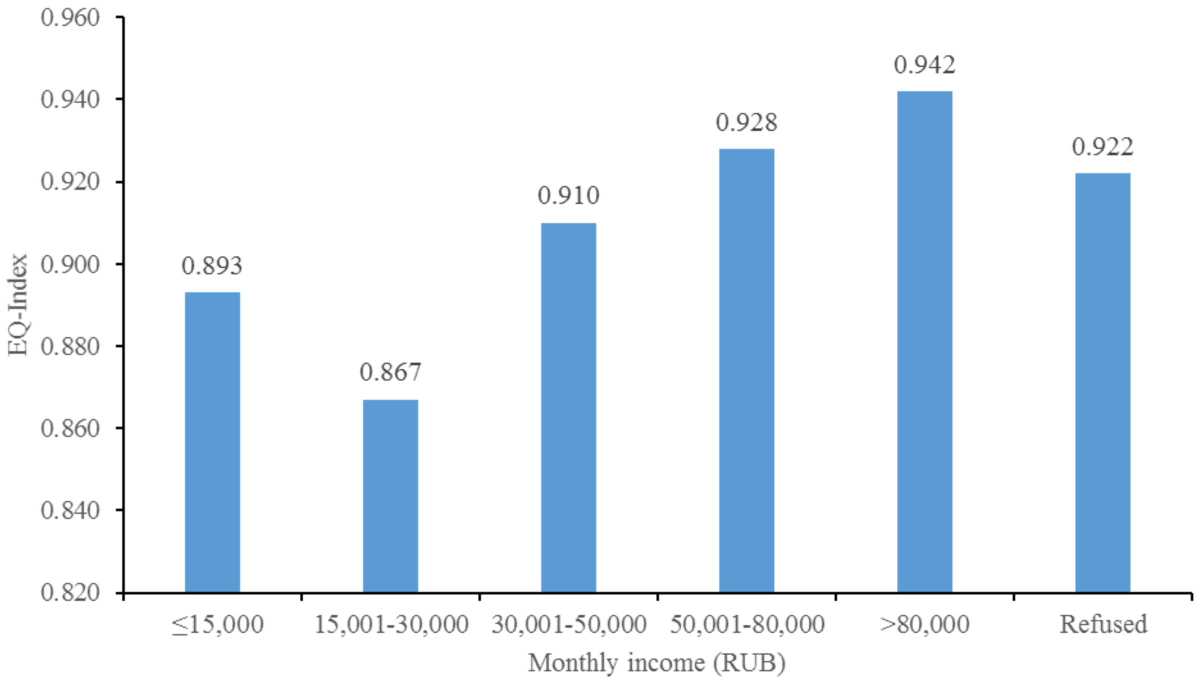

Supplemental Fig. 6 Any problems (%) within EQ-5D-5L dimensions according to Internet usage

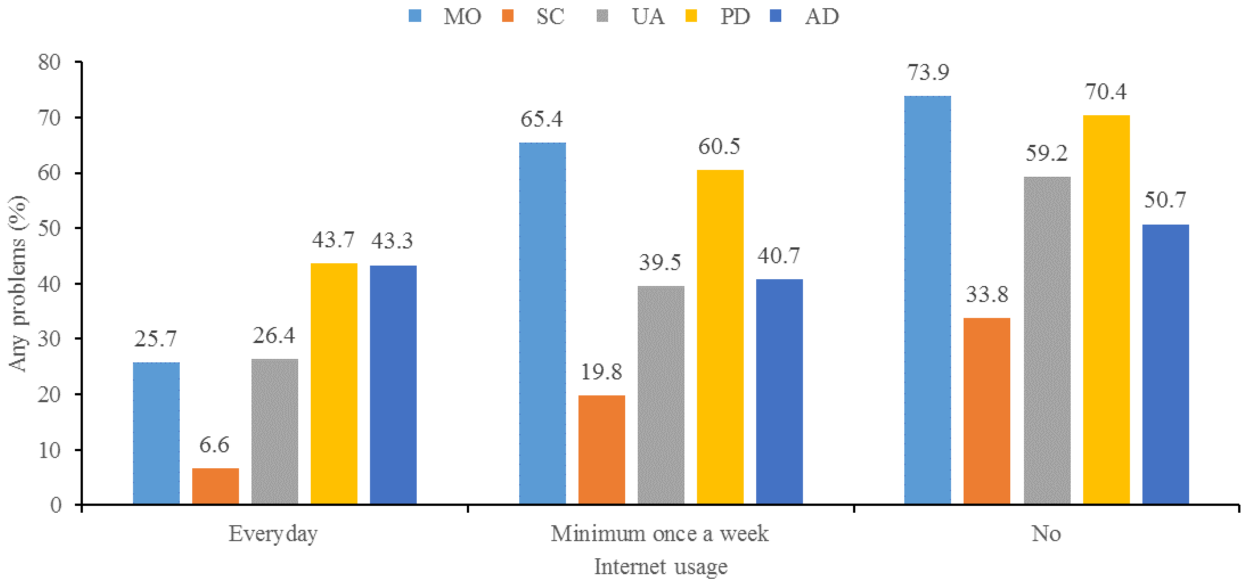

Supplemental Fig. 7 EQ VAS according to Internet usage

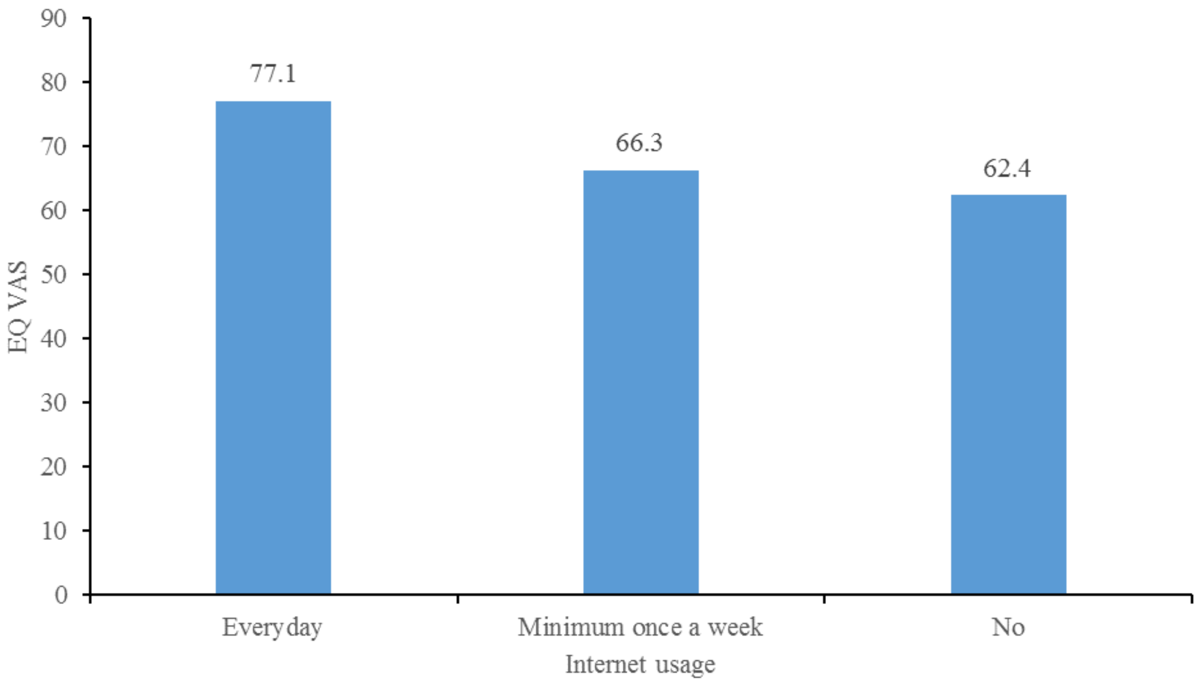

Supplemental Fig. 8 EQ-5D-5L index according to Internet usage

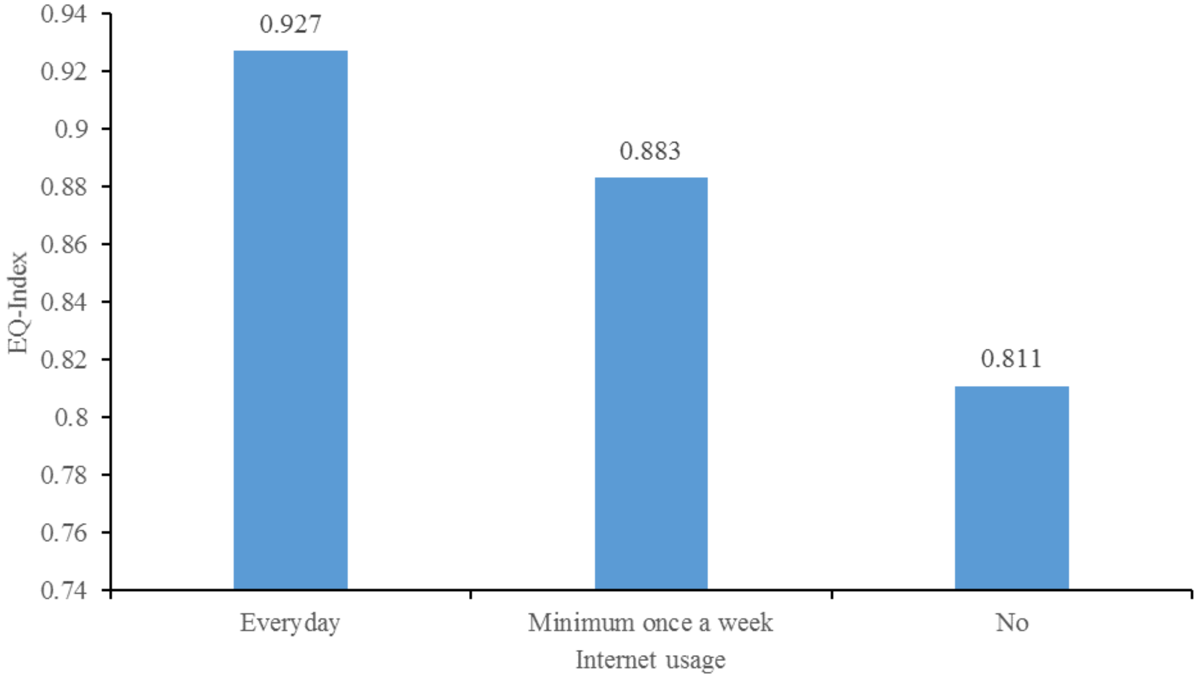

Supplement: Supplementary file 1 — Supplementary file1 (PDF 424 KB) [file 11136_2020_2705_MOESM1_ESM.pdf]
